# Cross Acclimation between Heat and Hypoxia: Heat Acclimation Improves Cellular Tolerance and Exercise Performance in Acute Normobaric Hypoxia

**DOI:** 10.3389/fphys.2016.00078

**Published:** 2016-03-08

**Authors:** Ben J. Lee, Amanda Miller, Rob S. James, Charles D. Thake

**Affiliations:** ^1^Department for Health, University of BathBath, UK; ^2^Centre for Applied Biological and Exercise Sciences, Coventry UniversityCoventry, UK

**Keywords:** heat, hypoxia, cross-acclimation, cycling, heat shock proteins

## Abstract

**Background:** The potential for cross acclimation between environmental stressors is not well understood. Thus, the aim of this investigation was to determine the effect of fixed-workload heat or hypoxic acclimation on cellular, physiological, and performance responses during post acclimation hypoxic exercise in humans.

**Method:** Twenty-one males (age 22 ± 5 years; stature 1.76 ± 0.07 m; mass 71.8 ± 7.9 kg; $\dot{\text{V}}$O_2_ peak 51 ± 7 mL^.^kg^−1.^min^−1^) completed a cycling hypoxic stress test (HST) and self-paced 16.1 km time trial (TT) before (HST1, TT1), and after (HST2, TT2) a series of 10 daily 60 min training sessions (50% N $\dot{\text{V}}$O_2_ peak) in control (CON, *n* = 7; 18°C, 35% RH), hypoxic (HYP, *n* = 7; fraction of inspired oxygen = 0.14, 18°C, 35% RH), or hot (HOT, *n* = 7; 40°C, 25% RH) conditions.

**Results:** TT performance in hypoxia was improved following both acclimation treatments, HYP (−3:16 ± 3:10 min:s; *p* = 0.0006) and HOT (−2:02 ± 1:02 min:s; *p* = 0.005), but unchanged after CON (+0:31 ± 1:42 min:s). Resting monocyte heat shock protein 72 (mHSP72) increased prior to HST2 in HOT (62 ± 46%) and HYP (58 ± 52%), but was unchanged after CON (9 ± 46%), leading to an attenuated mHSP72 response to hypoxic exercise in HOT and HYP HST2 compared to HST1 (*p* < 0.01). Changes in extracellular hypoxia-inducible factor 1-α followed a similar pattern to those of mHSP72. Physiological strain index (PSI) was attenuated in HOT (HST1 = 4.12 ± 0.58, HST2 = 3.60 ± 0.42; *p* = 0.007) as a result of a reduced HR (HST1 = 140 ± 14 b.min^−1^; HST2 131 ± 9 b.min^−1^ *p* = 0.0006) and T_rectal_ (HST1 = 37.55 ± 0.18°C; HST2 37.45 ± 0.14°C; *p* = 0.018) during exercise. Whereas PSI did not change in HYP (HST1 = 4.82 ± 0.64, HST2 4.83 ± 0.63).

**Conclusion:** Heat acclimation improved cellular and systemic physiological tolerance to steady state exercise in moderate hypoxia. Additionally we show, for the first time, that heat acclimation improved cycling time trial performance to a magnitude similar to that achieved by hypoxic acclimation.

## Introduction

Adaptation to one environmental stressor can induce protective responses upon exposure to other stressors as long as they share common adaptive responses (Fregly, 2011). This phenomenon is termed cross-acclimation, when physiological strain is attenuated (Ely et al., 2014), or cross-tolerance, when improved cellular protection is observed (Kregel, 2002). At a cellular level acute heat (Fehrenbach et al., 2001; Lee et al., 2014; Périard et al., 2015) and hypoxic stress (Taylor et al., 2011; Lee et al., 2014) induce the heat shock response (HSR; Morimoto, 1998), characterized by a transient post exposure increase in the cytoprotective heat shock protein 72 (HSP72). Additionally, acclimation to either heat or hypoxia induces phenotypic alterations that increased expression of genes encoding for cytoprotective HSPs (Maloyan et al., 1999; Mcclung et al., 2008; Gibson et al., 2015c), leading to greater cellular resilience in the face of subsequent stressful insults (Levi et al., 1993; Hutter et al., 1994).

Enhanced HSP72 following heat acclimation is associated with delayed tissue injury during acute heat stress (Horowitz et al., 2004) and contributes to cross tolerance between heat and ischemic stressors (Maloyan et al., 1999). In humans supplemented with quercetin—a potent inhibitor of the heat shock response, post heat acclimation thermotolerance was reduced (Kuennen et al., 2011). This was characterized by a diminished cellular stress marker response alongside an attenuated physiological adaptation, illustrating the functional role of the HSR at a whole body level. However, Hom et al. (2012) demonstrated a heat acclimated phenotype in the absence of HSP72 elevation, suggesting that increased HSP72 alone may not always be present in heat acclimation. In rodent models, increased hypoxia-inducible factor 1-α (HIF1-α), the master regulator of oxygen-regulated genes, and downstream indicators of HIF-1α expression such as erythropoietin (EPO) receptor, EPO, and vascular endothelial growth factor (VEGF), have been observed after acute heat stress (Na'ama et al., 2005) and heat acclimation (Maloyan et al., 2005; Tetievsky et al., 2008; Assayag et al., 2012; Umschweif et al., 2013) suggesting an interaction between HIF-1α and the HSR during heat acclimation.

Few studies have investigated the potential for heat acclimation to confer cross acclimation and tolerance to acute hypoxic exposures in a human model (Heled et al., 2012; Lee et al., 2014, 2015; Gibson et al., 2015c), with no study examining the response ofHIF1-α to a heat acclimation regimen. Both acute exercising exposures to heat and heat combined with hypoxia have been shown to attenuate physiological strain during hypoxic exercise conducted 24 h following the initial heat exposure (Lee et al., 2014). The same authors also observed reduced exercise heart rates and rectal temperatures, and increased exercise SpO_2_ during hypoxic exercise that was preceded by 3 days of heat acclimation (Lee et al., 2015). Longer term heat acclimation programs have also led to a reduction in hypoxic exercise HR alongside increased SpO_2_ (Heled et al., 2012; Gibson et al., 2015c), and an improved cardiac efficiency ($\dot{\text{V}}$O_2_/HR referred to as O_2_ pulse; Gibson et al., 2015c). In addition to the improvements seen in systemic function, an increase in resting peripheral blood mononuclear cell (PBMC) HSP72 protein (Lee et al., 2014, 2015) and HSP72 mRNA (Gibson et al., 2015c) have been observed following heat acclimation. Subsequently, the post hypoxic exercise induced increases in the HSP72 response have been shown to be attenuated in heat-acclimated individuals (Lee et al., 2015; Gibson et al., 2015c). These data support the existence of both cross-acclimation and cross-tolerance in humans.

While each of these studies included matched load control groups (Lee et al., 2015; Gibson et al., 2015c), no study has examined cross-acclimation and cross-tolerance in relation to a matched period of hypoxic acclimation. Neither has the effect of heat acclimation on hypoxic exercise performance been determined. Therefore, the aim of the present study was to compare the impact of a period of heat acclimation vs. hypoxic acclimation on physiological cross-acclimation and cellular cross-tolerance, and exercise performance during a subsequent exposure to acute normobaric hypoxia. It was hypothesized that a prior period of either heat or hypoxic acclimation would reduce physiological strain and improve physical performance when exercising in moderate normobaric hypoxia, with the effects being greatest following hypoxic acclimation. It was also hypothesized that both heat and hypoxic acclimation would increase resting levels of both mHSP72 and eHIF-1α post acclimation.

## Methods

### Participant characteristics

**Figure 1:**
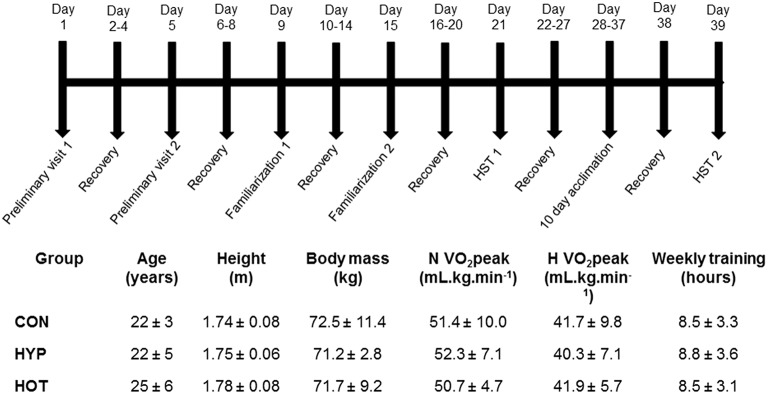
**Schematic of the experimental design, anthropometric and physiological characteristics of participants, indicating the typical days on which specific tests were undertaken**.

Participants (*n* = 21 males; Figure 1) provided written informed consent to take part in the study, which was approved by the Coventry University Ethics review panel. Established confounding variables of smoking, caffeine, glutamine, quercetin, alcohol, and prior thermal, hypoxic and hyperbaric exposures were all controlled in line with previous work (Taylor et al., 2011; Gibson et al., 2014; Lee et al., 2014, 2015). Participants were asked not to undertake any other exercise training in the 72 h leading up to a testing bout and throughout the intervention period. All data collection was conducted in accordance with the standards set out in the Declaration of Helsinki of 1996.

### Experimental design

All participants attended the laboratory on 17 separate occasions, as outlined in Figure 1. Two preliminary visits enabled height, body mass, estimated body fat via skinfold measurements, and normoxic (N) and hypoxic (H) $\dot{\text{V}}$O_2_ peak tests, separated by at least 5 days, to be conducted. Thereafter participants were split into three experimental groups (control, CON; heat acclimation, HOT; hypoxic acclimation, HYP) that were matched for H $\dot{\text{V}}$O_2_ peak and training experience (Figure 1). After both N and H $\dot{\text{V}}$O_2_ peak tests had been conducted all participants undertook two hypoxic stress test (HST) familiarization (FAM) sessions (described below) at least 4 days apart (Lee et al., 2014, 2015). To avoid any confounding acclimation to acute hypoxia FAM was conducted under normoxic conditions. At least 7 days after the final FAM session participants completed the first HST.

### Preliminary visit measurements

Height was measured in the Frankfurt plane using a Harpenden stadiometer (Harpenden Instruments, Burgess Hill, UK), nude body mass determined on an electronic scale (Seca Body, Cranlea and Company, Birmingham, UK) and sum of skinfolds determined from four sites using a skinfold caliper (Harpenden Instruments, Burgess Hill, UK) as described by Durnin and Womersley (1974).

Peak $\dot{\text{V}}$O_2_ was determined in both N and H conditions on separate days (preliminary visits 1 and 2) using an incremental exercise test to volitional exhaustion on a calibrated SRM cycle ergometer (Schoberer Rad Meßtechnik, Welldorf, Germany). Hypoxia was generated via a Hypoxicator unit (Hypoxico HYP123 Hypoxicator, New York, USA), that was used to fill a reservoir of three 1000 L Douglas bags in series. Participants inspired via a mouthpiece attached to a two-way non-rebreathable valve (Harvard Ltd., Eldenbridge, UK) connected to the gas reservoir with clear ethylene vinyl tubing.

Resting blood lactate (BLa; Biosen C-Line analyser, EKF Diagnostics, Sailauf, Germany) was determined from a finger capillary whole blood sample following a 10-min seated rest period. The test began at a workload of 70 W for 4 min and was then increased by 35 W every 4 min until a blood lactate value of >4 mmol·L^−1^ was reached. Thereafter, workload increased 35 W every 2 min until volitional exhaustion. A cadence of 70 rev·min^−1^ was maintained throughout. Expired gases were collected using 200 L Douglas bags (Cranlea & Co, Birmingham, UK) during the final minute of each stage. Heart rate (Polar FT1, Polar Electro OY, Kempele, Finland) and perceived exertion (Borg, 1982) were recorded at the end of each gas collection. Respiratory gas analysis was completed as previously described (Lee et al., 2014, 2015).

### Familiarization, hypoxic stress testing (HST), and acclimation procedures

On each FAM and HST session, as well as throughout the acclimation period, participants reported to the laboratory after an overnight fast to consume a set breakfast 2 h prior to the exercise bout. The energy content of the breakfast was 386 kcal, made up of 15.6 g protein, 44.4 g carbohydrate and 16.4 g fat. Participants drank 400 ml of water with the breakfast.

Each FAM, HST and acclimation session was preceded by 15 min of seated normoxic rest (after instrumentation) to collect baseline data and an additional 15 min of seated rest within the defined environment (N or H). The FAM and HST sessions consisted of 40 min of cycle exercise at 50% N $\dot{\text{V}}$O_2_ peak, a 5 min recovery in which instruments were removed from participants, followed by a 16.1 km cycling time trial. The time trial has a CV and TEM of 0.63% and 36 s, respectively, following two FAM sessions. The smallest worthwhile change in TT time using this protocol is therefore a 46 s difference (Lee et al., 2015). The 10-day acclimation protocol consisted of once daily exposures of cycle ergometer exercise within the defined environment, either CON (18°C, 35% RH), HOT (40°C, 25% RH), or HYP (18°C, 35% RH, F_I_O_2_ = 0.14%) at 50% N$\;\dot{\text{V}}$O_2_ peak for 60 min (Castle et al., 2011).

### Physiological measurements

Prior to each testing session participants provided a urine sample for the assessment of urine specific gravity (USG; Atago Refractomer, Jencons Pls, Leighton Buzzard, UK) and urine osmolality (U_OSMO_; Advanced 3300 Micro-Osmometer, Advanced Inc., Massachusetts, USA), determined their nude body mass (Seca, Bodycare, UK) and inserted a rectal thermistor (Grant Instruments, UK) to a depth of 10 cm. Heart rate (HR) was monitored throughout each trial via telemetry (Suunto, T6c, Finland). Blood lactate (Biosen C-Line analyser, EKF Diagnostics, Sailauf, Germany) was determined from a finger capillary whole blood sample at the end of the resting period and at the end of exercise for both HST and acclimation sessions. Heat strain was calculated using the physiological strain index (PSI; (Moran et al., 1998) as follows:

$$\begin{array}{l} \text{PSI}=5({\text{T}}_{\text{rectalT}}-{\text{T}}_{\text{rectal}0})\times {\left(39.5-{\text{T}}_{\text{rectal}0}\right)}^{-1}+5({\text{H}}_{\text{RT}}-\\ \;{\text{HR}}_{0})\times {\left(180-{\text{HR}}_{0}\right)}^{-1} \end{array}$$

Where T_rectal0_ and HR_0_ are the initial T_rectal_ and heart rate respectively, and T_rectalT_ and HR_T_ were obtained at 10 min intervals during acclimation sessions or HST with the mean exercise value reported, and at each 1 km measurement point throughout the TT. The PSI classifies physiological strain between 0 and 10 units, where 0 represents no or very little strain and 10 represents very high strain (Moran et al., 1998).

During all hypoxic sessions, arterial oxygen hemoglobin saturation (S_P_O_2_) was measured throughout via a pulse oximeter (WristOx, Nonin Medical Inc., Minnesota, USA). Hemodynamic indices of stroke volume (SV) and cardiac output ($\dot{\text{Q}}$) were estimated via arterial waveform measurements obtained from a pneumatic finger cuff attached to the index finger of the right hand (Portapres Model-2, Finapres Medical Systems, Hogehilweg, Amsterdam). The right arm was supported throughout using a sling, and the index finger positioned at a height equivalent to the aorta via palpation of the third intercostal space. Measurements were taken at the end of each resting phase, and for 120 s every 10 min during the exercise phase, and Portapres calibrations performed at the beginning of the rest period and at 8 min intervals throughout the HST. Ratings of perceived exertion (RPE; Borg, 1982) and thermal sensation (TS) were collected at 10 min intervals during the 40 min exercise tolerance phase of the test session with the mean exercise value reported.

The self-paced 16.1 km time trial was completed using the SRMwin software's open-ended mode (Version 6.4.2). Participants were instructed to complete the course as quickly as possible and were given no verbal encouragement during the TT. Participants were only able to see the distance they had covered. Measures of HR, SpO_2_, T_rectal_, and power output were collected every kilometer and a fingertip BLa sample collected immediately upon completion of the TT.

### Blood sampling

Venous blood samples (~7 mL) were collected from an antecubital vein into an EDTA treated vacutainer (Vacuetter, Greiner Bio-One, Stonehouse, UK) following the 15 min seated stabilization period before each HST, and on day 1 and 10 of the acclimation period. Post exercise samples were collected immediately after the exercise phase of the 60 min HST exposure was completed, and immediately upon completion of the aforementioned acclimation sessions. Whole venous blood was used to determine hemoglobin via a calibrated B-Hemoglobin Photometer (Hemocue Ltd., Angleholm, Sweden) and heparinized capillary sample tubes were centrifuged (Hawksley Micro Hematocrit Centrifuge, Hawksley and Son, Lancing, UK) to establish hematocrit using a micro hematocrit reader (Hawksley Micro). Both hemoglobin and hematocrit were assessed in triplicate with the mean value reported. These samples were collected to calculate corrected plasma volumes according to the equations of Dill and Costill (1974). A 100 μL aliquot was used for the immediate assessment of monocyte HSP72 (mHSP72; described below). The remaining whole blood was centrifuged at 5000 rpm for 10 min and plasma aliquots stored at −80°C until assessment of plasma glucose and lactate (Randox Daytona Rx, County Antrim, Ireland), and extracellular HIF-1α (eHIF-1α; Cusabio, BIOTEK, Newark, New Jersey).

### mHSP72 determination

An IgG1 isotype and concentration-matched FITC-conjugated negative control were used in order to assess non-specific binding. Briefly, cells obtained after red cell lysis were fixed and permeabilised (AbD Serotec, Kidlington, UK) and a negative control (FITC, AbD Serotec, Kidlington, UK) or anti-HSP72 antibody (SPA-810, Enzo lifesciences, Exeter, UK) was added to a final concentration of 100 μg·ml^−1^, this was used to label 1 × 10^6^ cells according to the manufacturer's instructions and then incubated for 30 min in the dark. Samples were then analyzed on a BD FACSCalibur (BD Biosciences, Oxford, UK) by flow cytometry with monocytes gated for forward/side scatter properties and further discriminated by CD14 expression (Selkirk et al., 2009). Mean florescence intensity (MFI) was then calculated using CellQuest Pro software (BD Biosciences, Oxford, UK) with a total of 15000 cells counted.

### Extracellular HIF-1α

Extracellular HIF-1α, in EDTA plasma, was measured using a pre-prepared sandwich enzyme-linked immunosorbent assay (ELISA) technique (Cusabio BIOTEK, Newark, New Jersey). 100 μL of standards and samples were added to each pre-coated well and incubated for 2 h at 37°C. Standards and samples were then aspirated and 100 μL of biotin-antibody and incubated at 37°C for 1 h. After three 200 μL washes with sodium azide-free wash buffer, 100 μL of horse radish peroxidase-avidin was added to each well and incubated for 1 h at 37°C. Following a further three wash steps 90 μL of TMB substrate was added and incubated at 37°C in the dark before 50 μL of stop solution was added. The plate was then read at 570 nm and 450 nm to enable wavelength correction. The assay's detection range is 62.5−4000 pg.mL^−1^ and limit of detection is 15.6 pg.mL^−1^. The intra-assay precision was determined from duplicates of standards within the same plate (6.4%) and inter-assay precision determined from standards assessed across plates (8.2%).

### Statistical analysis

The primary outcome measures of this study were an assessment of whole body cardiovascular, thermoregulatory, metabolic and mHSP72, and eHIF-1α responses to the HST. Time trial completion time was the main variable of interest during the performance component of the HST. In order to control for the false discovery rate and correct for multiple comparisons four families of hypothesis were tested according to the method of Benjamini and Hochberg (1995); (1) Physiological responses to acclimation; (2) Physiological responses to the hypoxic stress tests; (3) Cellular stress responses; and (4) Time trial performance responses.

All data were checked for normal distribution prior to analysis and tests employing repeated measures were checked for sphericity before analysis with Mauchly's sphericity test. Where sphericity was broken, *p*-values were corrected using the Huynh-Feldt method. Resting and mean exercise data from day 1 and day 10 of the acclimation period, and HST1 and HST2 were analyzed using a 2 (time) × 3 (group) mixed effects linear model, with fixed effects for acclimation day.

To enable an exploration of pacing strategies during the TT following the acclimation period power output was averaged over each km of the TT and analyzed using linear effects mixed models with fixed effects for time and group. HR, T_rectal_ and PSI during the TT were analyzed using the same method. Data are reported as mean ± standard deviation for *n* = 7 in each experimental group, unless otherwise stated. Precise *p*-values are reported, and both Cohen's D (with 95% confidence intervals) and partial eta squared (Pη^2^) effect sizes are presented to indicate the magnitude of observed effects (Colquhoun, 2014). Cohens D effect sizes of 0.2, 0.5, and 0.8 and partial eta squared (Pη^2^) effect sizes of 0.01, 0.06, and 0.13 are considered small, medium and large, respectively.

## Results

### Heat acclimation, hypoxic acclimation, and exercise control interventions

Physiological and thermoregulatory variables for each experimental group before and after day 1 and day 10 of acclimation are displayed in Table 1. Participants were hydrated prior to each acclimation session, with no between-day or between group differences observed for pre-trial body mass, U_osmo_ or USG. On day 1 of acclimation, mean exercise HR was greater in HOT (*p* = 0.01) and HYP (*p* = 0.02) compared to CON. Additionally, HOT induced a greater mean exercise T_rectal_ and corresponding PSI compared to HYP (both *p* < 0.05) and CON (both *p* < 0.001), with no difference observed between HYP and CON (Table 1).

**Table 1:** **Cardiovascular, thermoregulatory and perceptual responses at rest and during exercise for day 1 and day 10 of the acclimation period**.

	**Day 1**			**Day 10**		
**Variable**	**CON**	**HYP**	**HOT**	**CON**	**HYP**	**HOT**
USG	1.004 ± 0.01	1.020 ± 0.01	1.020 ± 0.01	1.010 ± 0.01	1.010 ± 0.01	1.020 ± 0.01
U_OSMO_ (mOsms^.^kg^−1^)	353 ± 254	465 ± 239	444 ± 234	320 ± 105	331 ± 176	463 ± 217
Hemoglobin (g/dL^−1^)	15.1 ± 0.9	14.9 ± 0.6	15.5 ± 1.0	14.9 ± 1.0	15.4 ± 0.4	14.8 ± 0.9^*^
Haemotocrit	0.44 ± 0.01	0.45 ± 0.02	0.44 ± 0.02	0.44 ± 0.02	0.43 ± 0.02	0.45 ± 0.01
Plasma volume (%)	55.8 ± 1.2	55.6 ± 2.3	55.9 ± 2.3	56.9 ± 2.0	53.3 ± 1.1	59.3 ± 2.2^#^
HR rest (bts^.^min^−1^)	71 ± 11	71 ± 13	78 ± 15	70 ± 10	71 ± 14	67 ± 11
T_rectal_ rest (°C)	37.04 ± 0.19	37.09 ± 0.18	37.19 ± 0.12	37.09 ± 0.12	36.96 ± 0.21	36.93 ± 0.26^#^
Mean HR (bts^.^min^−1^)	135 ± 11	151 ± 13^+^	151 ± 11^^^	135 ± 12	137 ± 9^#^	137 ± 12^*^
Mean T_rectal_(°C)	37.76 ± 0.25	37.86 ± 0.45	38.26 ± 0.11^+^^ψ^	37.73 ± 0.22	37.70 ± 0.21	37.72 ± 0.18^#^
Mean PSI (AU)	4.39 ± 0.84	5.14 ± 0.78	5.91 ± 0.66^^^^ψ^	4.26 ± 0.85	4.35 ± 0.61^#^	4.62 ± 0.48^#^
Δ T_rectal_(°C)	0.72 ± 0.30	0.77 ± 0.44	1.08 ± 0.17^^^^ψ^	0.63 ± 0.25	0.77 ± 0.25	0.78 ± 0.22^*^
Δ Body mass (kg)	0.76 ± 0.36	0.84 ± 0.38	1.01 ± 0.60^^^^ψ^	0.70 ± 0.31	0.87 ± 0.34	1.90 ± 0.31^^^^#^^ψ^
Mean RPE	12 ± 1	12 ± 2	12 ± 2	11 ± 1	10 ± 2	11 ± 2
Mean TS	4.8 ± 0.2	4.7 ± 0.6	6.3 ± 0.4^+^^ψ^	4.4 ± 0.5	4.1 ± 0.6	5.4 ± 0.4^*^

Data are means ± SD for all 21 participants.

* Different from acclimation day 1 (p < 0.05) within group.

# Different from acclimation day 1 (p < 0.01) within group.

+ Different from CON (p < 0.05).

^ Different from CON p (< 0.01).

ψ Different from HYP (p < 0.05).

On day 10 of acclimation an interaction effect was observed for resting plasma volume (*f* = 12.336, *p* < 0.0001, ηp^2^ = 0.58) which increased in HOT (*d* = 3.8, 95% CI = 1.9 to 5.2), decreased in HYP (*d* = −1.7. 95% CI = −3.2 to −0.6) and remained unchanged in CON (*d* = 0.7, 95% CI = −0.5 to 1.7). No main effect for time (*f* = 3.346, *p* = 0.084, ηp^2^ = 0.16) or time × group interaction (*f* = 2.293, *p* = 0.13, ηp^2^ = 0.20) was observed for resting HR, although a large effect size was noted for HOT (*d* = −0.73, 95% CI = −1.86 to 0.31). Resting T_rectal_ was lower following acclimation in both HYP (*d* = −0.72, 95% CI = −1.69 to 0.45) and HOT (*d* = −2.17, 95% CI = −2.3 to –0.1; *p* < 0.05 for both).

Following acclimation PSI was reduced in both HYP (*p* = 0.001, *d* = −1.0, 95% CI = −2.2 to 0.1) and HOT (*p* = 0.005, *d* = −1.95, 95% CI = −3.4 to −0.8), and was unchanged in CON (*d* = −0.1, 95% CI = −1.2 to 0.9). The reduction in PSI was mediated by a lower mean exercise HR in HYP (*p* = 0.01, *d* = −1.1, 95% CI = −2.3 to 0.0), and a lower mean exercise HR (*p* = 0.04. *d* = −1.3, 95% CI = −2.3 to 0.0) and T_rectal_ (*p* = 0.001, *d* = 4.9, 95% CI = −5.0 to −1.7) in HOT. No change in these variables was observed in CON. A large effect for rate of T_rectal_ change was observed for HOT (*d* = −1.8, 95% CI = −2.6 to –0.3) and not for CON (*d* = −0.3, 95% CI = −1.4 to 0.8), or HYP (*d* = −0.1, 95% CI = −1.1 to 1.0), with no main effect for time (*F* = 4.240, *p* = 0.05, ηp^2^ = 0.19) or group × time interaction (*f* = 1.526, *p* = 0.244, ηp^2^ = 0.15) observed.

### Monocyte HSP72 before and after acclimation

**Figure 2:**
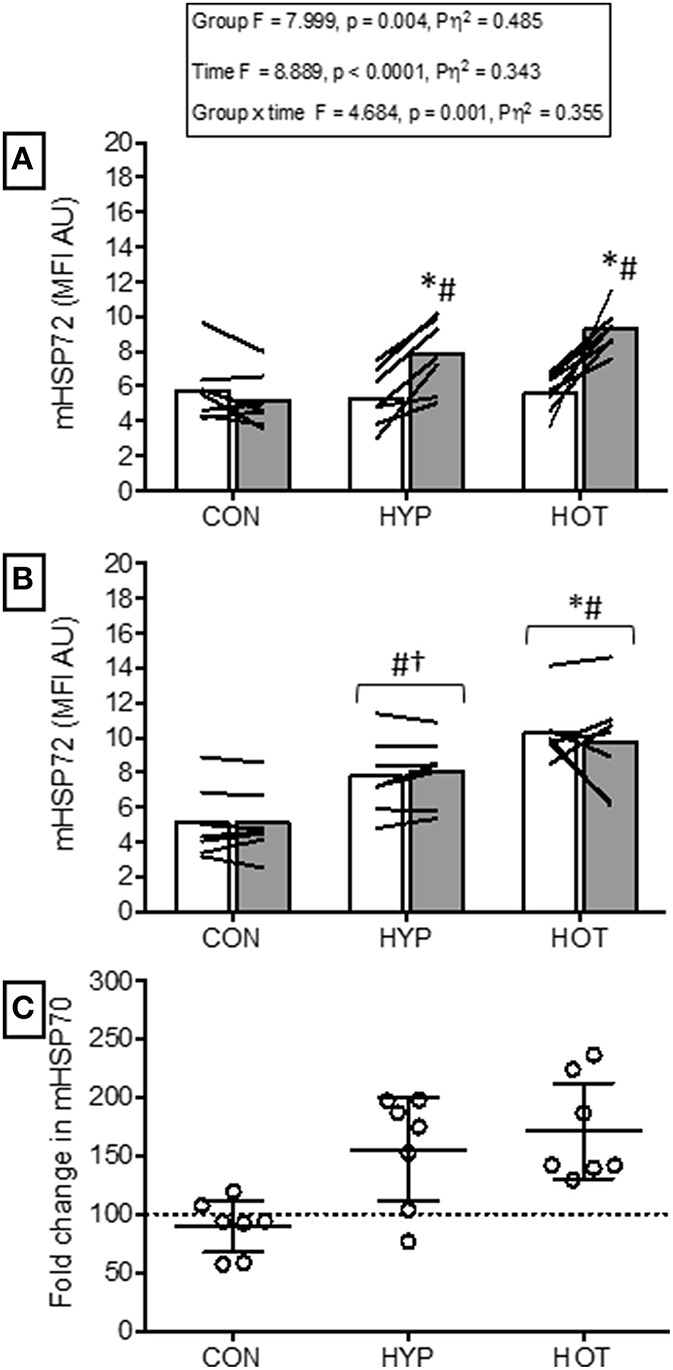
**Monocyte HSP72 responses before and after the acclimation period. (A)** mHSP72 is increased post exercise on day 1 of HYP and HOT, but not CON. (**B)** Resting mHSP72 was unchanged in CON and increased in HYP and HOT on day 10 of acclimation compared to day 1 of acclimation. Subsequently, the post exercise mHSP72 response in HYP and HOT was attenuated compared to post exercise on day 1. (**C)** The magnitude of change in resting mHSP72 on day 10 of acclimation was no different between HYP and HOT **(C)**. Open bars and shaded bars represent pre and post exercise, respectively. Lines (**A**, **B**) and dots **(C)** represent individual participant responses (*n* = 21) and bars show the mean group response. The dashed line **(C)** represents baseline mHSP72. ^*^Different from day 1 pre-exercise (*p* < 0.01). ^#^Different from control (*p* < 0.01). ^†^Different from control (*p* < 0.05).

No between group difference was observed for mHSP72 prior to acclimation (Figure 2A).

Following exercise on day 1, mHSP72 was increased in HOT (*p* = 0.0015, *d* = 3.8, 95% CI = 1.9 to 5.2) and HYP (*p* = 0.0009, *d* = 1.5, 95% CI = 0.1 to 2.4), but not CON (*p* = 0.14, *d* = −0.3, 95% CI = −0.5 to 0.7; Figure 2A). An inverse relationship between resting mHSP72 and the magnitude of after exercise expression was observed for HOT (*r* = −0.88, *p* = 0.009), with a weaker relationship observed for HYP (*r* = −0.52, *p* = 0.23).

Prior to day 10 of acclimation, resting mHSP72 was increased in HOT (*p* = 0.0006, *d* = 4.1, 95% CI = 1.4 to 4.4) and HYP (*p* = 0.013, *d* = 1.5, 95% CI = 0.04 to 2.3), and unchanged in CON (*p* = 0.21, *d* = −0.4, 95% = −1.4 to 0.7; Figure 2B). As a result of the increased resting concentrations of mHSP72, no further after exercise changes in mHSP72 were observed in HOT (*p* = 0.53) or HYP (*p* = 0.24) on day 10 (Figure 2B). Consequently, the before acclimation relationship between resting mHSP72 and magnitude in after exercise change was reduced in HOT (*r* = −0.05, *p* = 0.924), although similar in HYP (*r* = −0.55, *p* = 0.20).

### Extracellular HIF-1α before and after acclimation

**Figure 3:**
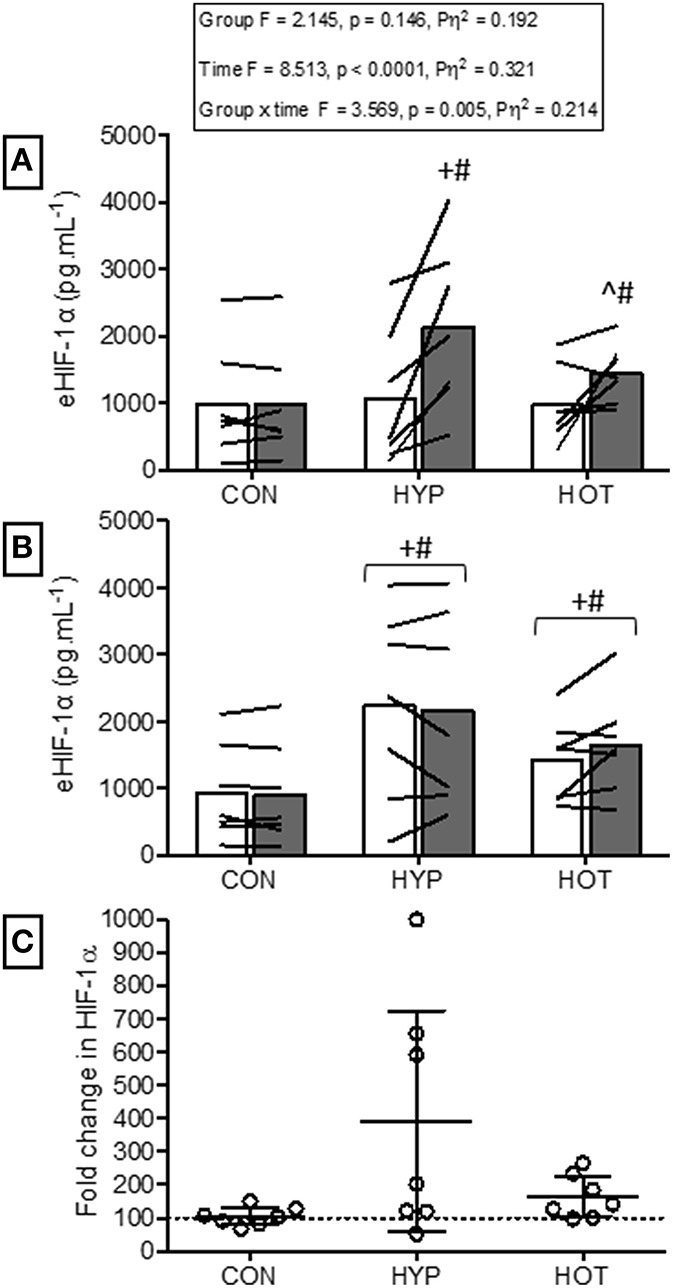
**Extracellular HIF-1α responses before and after the acclimation period. (A)** eHIF-1α is increased following an acute period of hypoxic exercise and is more variable following HOT. No post exercise changes in eHIF-1α were seen in CON (**B)** Resting eHIF-1α was elevated after 10 days of HYP and HOT acclimation, blunting the post exercise response on day 10 of acclimation. No changes in eHIF-1α were observed in CON. (**C)** The magnitude of change in resting eHIF-1α on day 10 of acclimation was no different between HYP and HOT. Open bars and shaded bars represent pre and post exercise, respectively. Lines (**A**,**B**) and dots **(C)** represent individual participant responses (*n* = 21) and bars the mean group response. The dashed line **(C)** represents baseline HIF-1α. ^+^Different from day 1 rest (*p* < 0.05). ^#^Different from control (*p* < 0.01). ^Different from day 1 pre exercise (*p* < 0.10). ^*^Different from control (*p* < 0.05).

Figure 3 illustrates eHIF1-α concentrations before and after day 1 and day 10 of acclimation. Following exercise on day 1 of acclimation eHIF-1α was increased in HYP (256 ± 290%, *p* = 0.011, *d* = 1.1, 95% CI = −0.2 to 2.0) and HOT (103 ± 162%, *p* = 0.076, *d* = 0.8, 95% CI = −0.4 to 2.0), and unchanged in CON (8 ± 29%, *p* = 0.95, *d* = 0.0, 95% = −1.1 to 1.0, Figure 3A). Prior to day 10 of acclimation eHIF-1α was increased in both HYP (292 ± 360%, *p* = 0.02, *d* = 1.2, 95% CI = −0.2 to 2.0) and HOT (165 ± 66%, *p* = 0.031, *d* = 0.80, 95% = −0.4 to 1.8), and unchanged in CON (5 ± 29%, *p* = 0.55, *d* = −0.1, 95% −1.1 to 1.0, Figure 3B). On day 10 of acclimation eHIF-1α was no different from rest after exercise in HYP (21 ± 79%, *p* = 0.628) HOT (19 ± 33%, *p* = 0.112) or CON (−5 ± 17%, Figure 3B).

### Hypoxic tolerance tests

Following acclimation and immediately prior to the beginning of the HST trial, plasma volume remained elevated in HOT (*p* = 0.022, *d* = 1.0, 95% CI = −0.3 to 1.9), depressed in HYP (*p* = 0.056, *d* = −1.1, 95% CI = −1.9 to 0.3) and unchanged in CON (*p* = 0.41, *d* = −0.2, 95% CI = −1.2 to 0.9). Resting physiological and thermoregulatory parameters are displayed in Table 2. No resting physiological variable was affected as a result of the intervention period (*p* > 0.05, Table 2). Table 3 presents the cardiovascular, metabolic, thermoregulatory, and perceptual responses to the HST before and after acclimation. HR was lower in HST2 compared to HST1 in HOT (*p* = 0.006, *d* = −0.6, 95% CI = −1.8 to 0.4), and unchanged in CON (*p* = 0.44, *d* = 0.2, 95% CI = Hypoxic Tolerance Tests 0.9 to 1.2) and HYP (*p* = 0.38, *d* = −0.1, 95% CI = −1.1 to 1.0). Moderate and large effects were observed for an increased SpO_2_ in HOT (*p* = 0.0015, *d* = 0.70 95% CI = −0.5 to 1.8) and HYP (*p* = 0.023, *d* = 0.50, 95% CI = −0.7 to 1.4), with no effect observed in CON (*p* = 0.36 *d* = 0.0), neither was an interaction effect observed (*f* = 1.69, *p* = 0.212). A moderate effect was observed for SV in HOT (*p* = 0.06, *d* = 0.40, 95% CI = −0.7 to 1.40) but no effect was observed for CON (*p* = 0.29, *d* = −0.1, 95% CI = −1.1 to 1.0) or HYP (*p* = 0.11, −0.4, 95% CI = −1.5 to 0.7), and no interaction effect was evident (*f* = 2.79, *p* = 0.08). As a result of the increased SV and decreased HR in HOT, and no changes in either component variable for CON and HYP, no interaction effect was observed for cardiac output (*f* = 0.50, *p* = 0.65), with small effects observed for CON (*d* = −0.1, 95% = −1.2 to 1.0), HYP (*d* = −0.4, 95% CI = −1.5 to 0.7), and HOT (*d* = −0.2, 95% CI = −1.3 to 0.8). Cardiac efficiency, as determined by O_2_ pulse, was improved in HOT (*p* = 0.01, *d* = 0.5, 95% CI = −0.6 to 1.5), and unchanged for CON (*p* = 0.50, *d* = 0.0, 95% CI = −1.1 to 1.1) and HYP (*p* = 0.34, *d* = −0.1, 95% CI = −1.12 to 0.98), although no interaction effect was observed (*f* = 3.32, *p* = 0.059). No differences were observed between HST1 and HST2 for $\dot{\text{V}}$E_min_, $\dot{\text{V}}$O_2_, $\dot{\text{V}}$CO_2_, or RER (Table 3).

**Table 2:** **Cardiovascular and thermoregulatory measurements at the end of the resting period during HST1 and HST2**.

	**HST 1**			**HST 2**		
**Variable**	**CON**	**HYP**	**HOT**	**CON**	**HYP**	**HOT**
Hemoglobin (g.dL^−1^)	15.2 ± 0.70	14.5 ± 0.3	15.5 ± 1.4	15.2 ± 0.9	15.2 ± 0.8	15.1 ± 1.3
Hematocrit (%)	0.44 ± 0.02	0.45 ± 0.01	0.46 ± 0.03	0.45 ± 0.02	0.44 ± 0.02	0.46 ± 0.02
Plasma volume (%)	55.2 ± 2.3	55.2 ± 2.31	53.9 ± 2.1	55.6 ± 2.1	52.7 ± 3.4^*^	55.8 ± 2.5^*^
HR (bts^.^min^−1^)	79 ± 8	81 ± 11	82 ± 16	82 ± 12	79 ± 18	79 ± 11
Plasma lactate (mmol^−1^)	1.51 ± 0.36	1.73 ± 0.52	1.55 ± 0.47	1.57 ± 0.41	1.44 ± 0.29	1.49 ± 0.57
Plasma glucose (mmol^−1^)	5.01 ± 0.97	4.81 ± 1.36	5.40 ± 0.99	4.87 ± 0.80	4.62 ± 0.52	4.54 ± 0.63
Stroke Volume (mL^.^bt^−1^)	79.9 ± 9.0	78.2 ± 14.8	78.1 ± 15.0	78.1 ± 11.5	77.2 ± 12.2	78.0 ± 18.2
Cardiac Output (L^.^min^−1^)	6.3 ± 1.0	6.3 ± 1.1	6.3 ± 1.1	6.4 ± 1.6	6.1 ± 1.5	6.2 ± 1.8
SpO_2_(%)	89 ± 2	89 ± 2	89 ± 3	89 ± 4	89 ± 3	91 ± 2
${\dot{\text{V}}}_{\text{E}}$ (L^.^min^−1^)	15.4 ± 4.0	13.7 ± 2.0	16.0 ± 2.5	15.4 ± 3.2	14.3 ± 1.6	16.5 ± 2.7
$\dot{\text{V}}$O_2_ (L^.^min-1)	0.36 ± 0.11	0.30 ± 0.07	0.36 ± 0.06	0.36 ± 0.11	0.32 ± 0.06	0.38 ± 0.12
T_rectal_(°C)	37.14 ± 0.15	37.02 ± 0.15	37.11 ± 0.20	37.19 ± 0.17	37.14 ± 0.16	37.08 ± 0.15

Data are means ± SD for all 21 participants.

* Different from HST1 (p < 0.05) within group.

**Table 3:** **Mean exercise cardiovascular, metabolic, thermoregulatory and perceptual measurements during HST1 and HST2**.

	**HST 1**			**HST 2**		
**Variable**	**CON**	**HYP**	**HOT**	**CON**	**HYP**	**HOT**
HR (bts^.^min^−1^)	139 ± 5	145 ± 15	140 ± 14	140 ± 8	144 ± 16	131 ± 9^#^
Stroke volume (mL^.^bt^−1^)	104 ± 12	112 ± 13	99 ± 10	103 ± 11	107 ± 8	103 ± 11
Cardiac output (L^.^min^−1^)	14.5 ± 1.8	16.1 ± 1.9	13.8 ± 1.3	14.3 ± 1.9	15.8 ± 1.4	13.5 ± 1.1
SpO_2_ (%)	82 ± 4	82 ± 2	83 ± 3	82 ± 3	83 ± 3^*^	85 ± 2^#^
Oxygen pulse (mL^.^bt^−1^)	12.6 ± 2.3	11.8 ± 1.3	11.5 ± 1.4	12.6 ± 2.1	11.7 ± 1.4	12.2 ± 1.4^*^
VE BTPS (L^.^min^−1^)	60.8 ± 10.4	63.2 ± 10.1	60.8 ± 5.0	59.2 ± 12.6	65.0 ± 9.0	58.8 ± 3.2
V°O_2_	1.76 ± 0.34	1.71 ± 0.26	1.60 ± 0.10	1.77 ± 0.35	1.68 ± 0.22	1.60 ± 0.14
V°CO_2_	1.69 ± 0.31	1.71 ± 0.23	1.57 ± 0.13	1.65 ± 0.38	1.66 ± 0.28	1.52 ± 0.12
RER	0.96 ± 0.06	1.00 ± 0.06	0.98 ± 0.06	0.93 ± 0.08	0.98 ± 0.09	0.95 ± 0.06
Plasma lactate (mmol^−1^)	3.44 ± 1.42	3.88 ± 2.08	3.25 ± 1.56	3.31 ± 1.39	3.03 ± 1.09	2.50 ± 0.87
Plasma glucose (mmol^−1^)	4.87 ± 1.13	5.07 ± 0.71	4.50 ± 0.98	4.65 ± 1.08	4.90 ± 1.16	4.77 ± 0.68
T_rectal_(°C)	37.61 ± 0.14	37.72 ± 0.18	37.55 ± 0.18	37.63 ± 0.11	37.69 ± 0.20	37.40 ± 0.14^#^
ΔT_rectal_(°C)	0.46 ± 0.19	0.70 ± 0.12	0.44 ± 0.36	0.44 ± 0.20	0.68 ± 0.20	0.32 ± 0.20^*^
PSI (AU)	4.2 ± 0.5	4.8 ± 0.6	4.1 ± 0.6	4.1 ± 0.6	4.8 ± 0.6	3.6 ± 0.4^#^
RPE	13 ± 1	12 ± 2	12 ± 2	12 ± 1	10 ± 2	11 ± 1
TS	3.9 ± 0.5	4.2 ± 0.8	4.3 ± 0.8	3.7 ± 0.4	4.0 ± 0.4	4.0 ± 0.4

Data are mean ± SD for all 21 participants.

* Different from HST1 to HST2 (p < 0.05) within group.

# Different from HST1 to HST2 (p < 0.01) within group.

An interaction effect was observed for T_rectal_ (*f* = 5.58, *p* = 0.013), with T_rectal_ lower during HST2 for HOT (*p* = 0.002, *d* = −0.6, 95% CI = −1.7 to 0.5), and unchanged for CON (*p* = 0.28, *d* = 0.1, 95% CI = −0.9 to 1.20) and HYP (*p* = 0.17, *d* = −0.2, 95% CI = −1.2 to 0.9). The attenuated HR and T_rectal_ observed in HOT resulted in a reduced PSI during HST2 (*p* = 0.007, *d* = −0.9, 95% CI = −2.1 to 0.2). PSI was unchanged from HST1 to HST2 in CON (*p* = 0.30, *d* = −0.1, 95% CI = −1.2 to 0.9) and HYP (*p* = 0.47, *d* = 0.02, 95% CI = −1.0 to 1.1). Additionally, the rate of T_rectal_ change was attenuated in HST2 following HOT (*p* = 0.026, *d* = −0.44, 95% CI = −1.4 to 0.7) but similar to HST1 in CON (*p* = 0.26, *d* = −0.1, 95% CI = −1.1*to*1.0) and HYP (*p* = 0.49, *d* = −0.2, 95% CI = −1.2 to 0.9).

### Monocyte HSP72 responses to acute hypoxia

**Figure 4:**
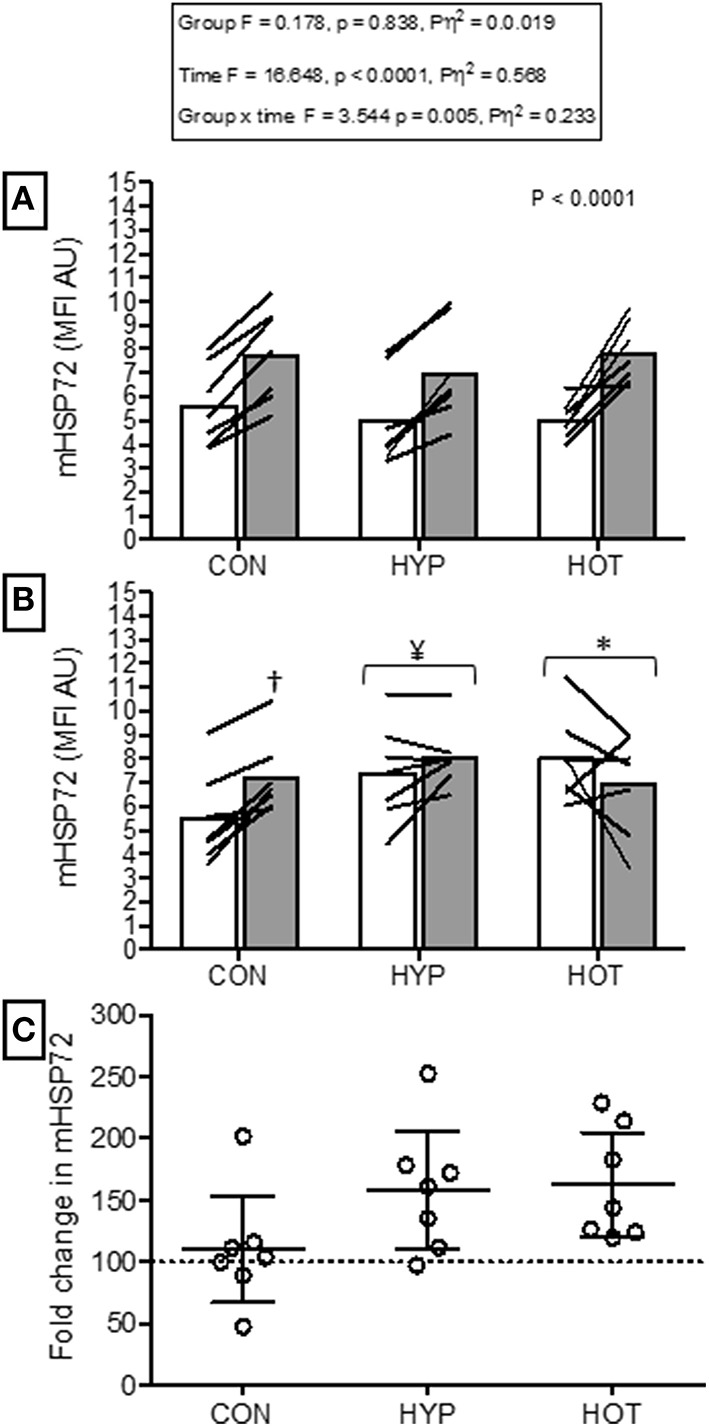
**Monocyte HSP72 before and after HST1 and HST2. (A)** mHSP72 is increased after a HST in 20 of 21 participants. (**B)** Resting mHSP72 was increased prior to onset of HST2 in HYP and HOT. The post exercise increase in mHSP72 was subsequently only observed in CON. (**C)** The magnitude of change in resting mHSP72 prior to HST2 was not different between HYP and HOT and were each elevated in comparison to CON. Lines (**A**,**B**) and dots **(C)** represent individual participant responses and bars the mean group response (*n* = 21). The dashed line **(C)** represents baseline mHSP72. ^†^Different from pre-exercise (*p* < 0.01). ^¥^Different from HST1 pre-exercise (*p* < 0.05). ^*^Different from HST1 pre-exercise (*p* < 0.01).

Figure 4 illustrates the mHSP72 response to hypoxia before (HST1) and after (HST2) the acclimation intervention. An acute bout of hypoxic exercise lead to increased mHSP72 MFI in all groups prior to acclimation (main effect for time, *F* = 16.65, *p* < 0.0001; Figure 4A), and the inverse relationship between resting mHSP72 and post exercise mHSP72 was observed (*r* = −0.51, *p* = 0.019 for the combined cohort, *n* = 21). Following acclimation resting mHSP72 was increased in HYP (58 ± 52%, *p* = 0.014, *d* = 1.2, 95% CI = −0.02 to 2.2) and HOT (63 ± 46%, *p* = 0.008, *d* = 3.8, 95% CI = 0.7 to 3.2), remaining unchanged in CON (10 ± 46%, *p* = 0.83, *d* = −0.1, 95% CI = −1.1 to 0.1; Figure 4B). Consequently, the mHSP72 response following HST2 was blunted for HOT (*p* = 0.26) and HYP (*p* = 0.18), and was comparable to HST1 in CON (Figure 4B).

### Extracellular HIF-1α responses to acute hypoxia

**Figure 5:**
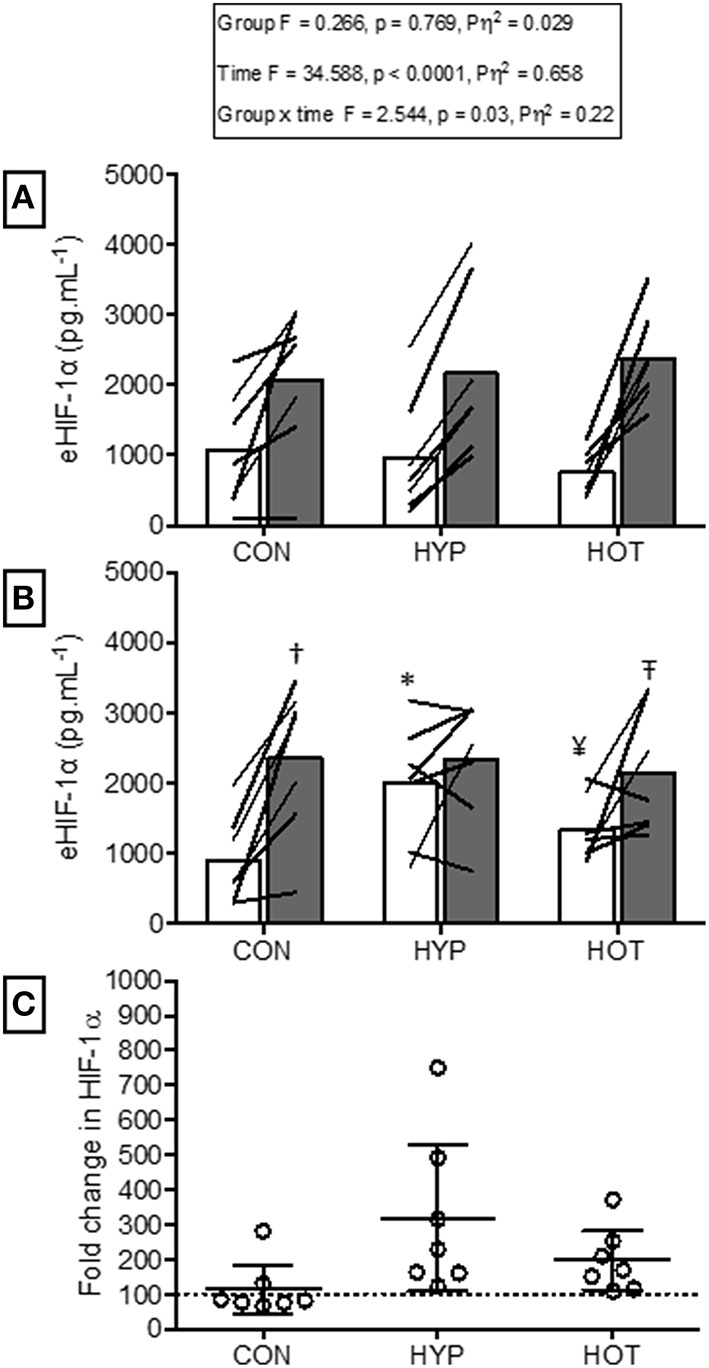
**Extracellular HIF-1α responses before and after HST1 and HST2. (A)** eHIF-1α increased in response to exercise in HST1 in all experimental groups. (B) Prior to HST2 resting levels of eHIF-1α were elevated in HYP when compared to pre HST1, and showed a varied individual response in HOT.eHIF-1α increased in response to exercise in HST2 in CON, but was unchanged in both HYP and HOT, although individual variation in the data is present. (**C)** The magnitude of change in resting eHIF-1α was not different between HYP and HOT prior to HST2, and each were elevated in comparison to CON. Lines (**A**,**B**) and dots **(C)** represent individual participant responses and bars the mean group response. The dashed line **(C)** represents baseline HIF-1α. Different from rest (*p* < 0.01) within trial. ^*^Different from HST1 pre-exercise (*p* < 0.01). ^¥^Different from HST1 pre-exercise (*p* < 0.05). – TDifferent from HST2 pre-exercise (*p* < 0.10). ^†^Different from control (*p* < 0.05).

Figure 5 illustrates the eHIF-1α response to hypoxia before (HST1) and after (HST2) the acclimation intervention. Prior to acclimation the HST induced a 171 ± 247%, 197 ± 125%, and 266 ± 192% increase in eHIF-1α in CON, HYP, and HOT, respectively (main effect for time, *F* = 34.59, *p* < 0.0001; Figure 5A). Following the 10 day acclimation period resting eHIF-1α was elevated in HYP (220 ± 128%, *p* = 0.002, *d* = 1.2, 95% CI = −0.2 to 2.0) and HOT (98 ± 92%, *p* = 0.017, *d* = 0.8, 95% CI = −0.4 to 1.8), and unchanged in CON (15 ± 76%, *p* = 0.62, *d* = 0.0, 95% CI = −1.1 to 1.0) (Figure 5B). Therefore, after acclimation eHIF-1α increased during exercise from rest in CON (241 ± 193%, *p* = 0.003, *d* = 2.3, 95% CI = 0.3 to 2.7) and HOT (76 ± 101%, *p* = 0.07, *d* = 1.8, 95% CI = −0.1 to 2.2), however this response was attenuated in HYP in comparison to HST1 (33 ± 83%, *p* = 0.30, *d* = 0.4, 95% CI = −0.7 to 1.4) (Figure 5B).

### Time trial performance

Table 4 illustrates performance changes for TT1 and TT2. There was no difference in TT times following the intervention in the CON group (TT1, 43:05 min:s, 95% CI = 40:18 to 45:51 min:s; TT2, 43:27 min:s, 95% CI = 40:54 to 45:58 min:s; *d* = 0.09). The HYP group were quicker in TT2 (41:32 min:s, 95% CI = 39:01 to 44:03 min:s) compared to TT1 (44:48 min:s, 95% CI = 42:02 to 47:33 min:s; *p* = 0.006, *d* = −1.14). The HOT group were also quicker in TT2 (40:41 min:s, 95% CI = 38:10 to 43:12 min:s) compared to TT1 (42:43 min:s, 95% CI = 39:58 to 45:29 min:s, *p* = 0.05, *d* = −0.70).

**Table 4:** **Individual data and mean ± *SD* data for performance variables during TT1 and TT2**.

	**Performance Time (mins)**		**Percent change (%)**	**Heart rate (bts.min**^**−1**^**)**		**T**_**rectal**_ **(**^**°**^**C)**		**PSI (AU)**	
	**TT1**	**TT2**		**TT1**	**TT2**	**TT1**	**TT2**	**TT1**	**TT2**
**CON**									
1	42.0	42.0	0.05	161	160	37.94	38.14	4.94	5.73
2	41.0	40.4	−1.39	159	165	37.73	38.10	5.52	6.20
3	51.2	50.6	−1.21	140	156	37.94	37.74	4.14	4.91
4	39.4	38.0	−3.58	178	183	38.22	38.23	6.62	7.57
5	41.4	44.0	6.18	148	153	38.03	38.61	4.79	6.14
6	41.5	41.0	−1.30	173	173	38.00	38.00	6.41	6.25
7	45.0	48.1	6.91	177	175	38.01	38.04	6.48	6.48
Mean + *SD*	43.1 ± 4.2	43.5 ± 4.3	0.8 ± 3.3	162 ± 15	166 ± 11	37.98 ± 0.1	38.1 ± 0.26	5.56 ± 0.97	6.18 ± 0.80
**HYP**									
8	43.0	42.4	−1.23	131	136	37.83	37.70	5.28	4.76
9	47.4	41.3	−12.97	127	126	37.80	37.95	4.84	6.58
10	41.3	39.3	−4.93	166	170	38.32	38.74	7.06	8.27
11	45.2	41.3	−8.52	154	154	37.94	37.97	5.46	5.94
12	41.5	39.1	−5.92	137	160	37.66	38.31	4.71	5.53
13	51.6	43.1	−16.28	158	157	38.25	38.25	5.57	6.49
14	43.4	44.1	1.47	171	173	38.03	38.12	5.83	6.19
Mean + *SD*	45.0 ± 4.0	41.5 ± 1.7	−6.9 ± 5.5	157 ± 13	162 ± 15	37.98 ± 0.24	38.15 ± 0.33	5.54 ± 0.78	6.25 ± 1.09
**HOT**									
15	39.0	37.3	−4.23	168	170	38.08	38.04	6.94	6.86
16	41.1	40.1	−2.43	174	175	38.05	37.92	6.37	6.27
17	43.4	42.0	−3.23	162	157	38.23	37.90	6.72	5.82
18	46.4	43.2	−6.87	149	147	38.03	37.93	4.88	5.37
19	45.4	44.2	−2.64	176	184	38.12	38.11	6.26	7.01
20	40.4	38.3	−5.29	151	156	37.80	37.80	4.31	5.35
21	43.2	39.5	−8.55	172	173	38.18	38.33	5.97	6.72
Mean + *SD*	42.7 ± 2.9	40.7 ± 2.8	−4.8 ± 1.7	164 ± 11	166 ± 13	38.07 ± 0.14	38.00 ± 0.17	5.92 ± 0.97	6.20 ± 0.70

**Figure 6:**
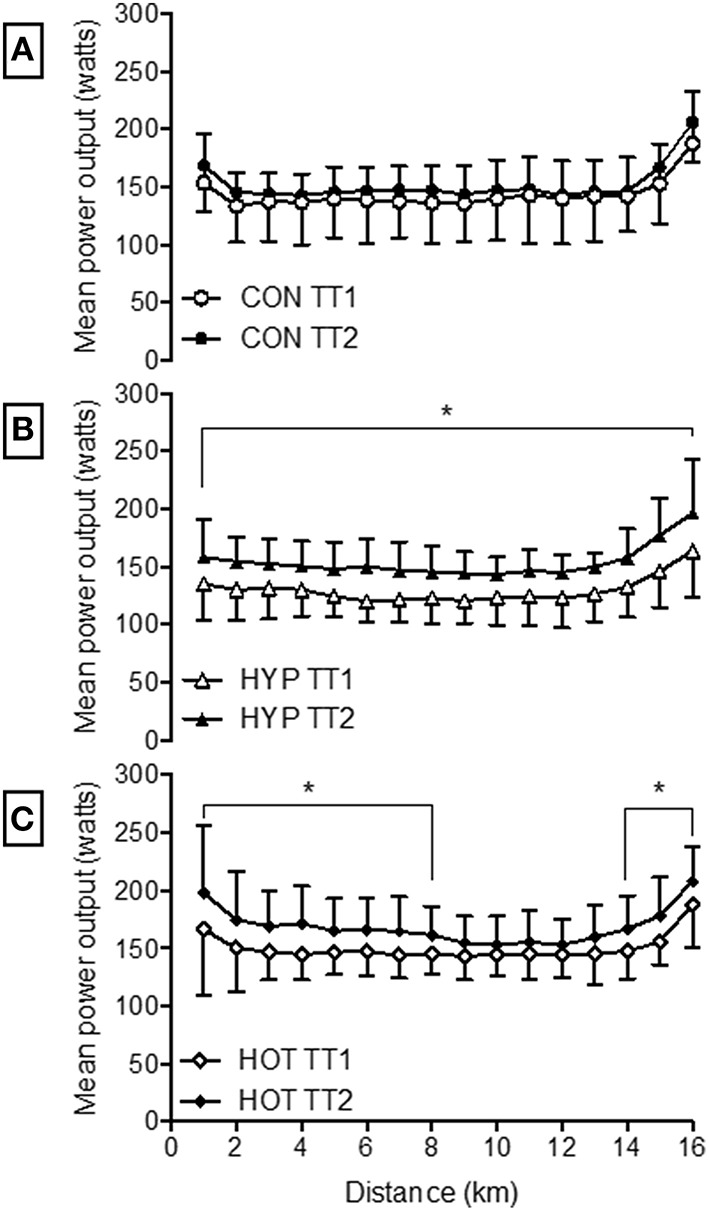
**Mean power output during each kilometer of the 16.1 km time trial for CON (A), HYP (B) and HOT (C)**. ^*^Difference from TT1 (*p* < 0.05).

Power output during TT2 was increased from TT1 in the HYP and HOT groups (*p* < 0.05, Figure 6). Specifically PO was greater during each kilometer in HYP (Figure 6B), and greater between 1–8 km and 14–16 km in HOT (Figure 6C). HR and T_rectal_ were no different during the TT pre to post intervention in any experimental group (*p* > 0.05, Table 4). PSI was higher in the post intervention TT in the CON (*p* = 0.02) and HYP groups (*p* = 0.03).

Upon completion of acclimation, HST2 and TT2, normoxic $\dot{\text{V}}$O_2_ peak was unchanged from pre-intervention values in all groups (CON: pre 51.4 ± 10.0 mL.kg^−1.^min^−1^, post 51.9 ± 8.6 mL.kg^−1.^min^−1^; HYP, pre 50.7 ± 4.7 mL.kg^−1.^min^−1^, post 51.4 ± 5.4; HOT, pre 52.3 ± 7.1 mL.kg^−1.^min^−1^, post 53.4 ± 6.5 mL.kg^−1.^min^−1^). Additionally, peak power output and power at lactate threshold also remained unchanged between groups upon completion of the experimental period.

## Discussion

### Summary of main findings

The main finding of this study is that fixed-work heat acclimation reduces physiological strain, as assessed by exercising HR and T_rectal_, and improves cycling TT performance in acute normobaric hypoxia. Moreover this effect was comparable to the decreased physiological strain and enhanced performance achieved with hypoxic acclimation and occurred despite no post acclimation improvement in $\dot{\text{V}}$O_2_ peak or lactate threshold in any experimental group. Therefore, the hypothesis pertaining to heat acclimation resulting in improved systemic hypoxic tolerance is accepted. At a cellular level both HOT and HYP increased resting mHSP72 prior to the second HST, supporting the hypothesis that both heat and hypoxic acclimation would enhance cellular tolerance. As a result the cellular stress response to hypoxia was blunted in the HYP and HOT groups. Interestingly, eHIF-1α was elevated in both HYP and HOT immediately post exercise after the initial acclimation session. Thereafter an increased resting concentration was only noted following HYP (48 h post HST). Increased baseline eHIF-1α led to a blunted post hypoxic exercise eHIF-1α response in the HYP group, whereas data from the HOT group was equivocal, indicating that further study on eHIF-1α and related downstream markers regulated by this oxygen sensing gene following heat acclimation are warranted.

### Heat and hypoxic cross-acclimation

Heat acclimation induced a greater adaptive stimulus at lower levels of metabolic strain, and in a shorter time frame compared to hypoxic acclimation. This occurred despite the HYP group completing sessions at a higher relative intensity (61 ± 0.5% of H $\dot{\text{V}}$O_2_ peak). Exercise durations of up to 90 min, as frequently utilized in acclimation protocols (De Castro Magalhães et al., 2010; Gibson et al., 2015b,c) increase the variability in trial duration between hypoxia and heat stress (Lee et al., 2014). Therefore, matching both cardiovascular strain and exercise duration during the initial phase of acclimation was achieved by using a workload of 50% N $\dot{\text{V}}$O_2_ peak for 60 min (Lee et al., 2014). While cardiovascular strain in the HOT and HYP groups were each higher than CON during the initial phases of acclimation, the total physiological strain was greatest in the HOT group as a result of the elevated T_rectal_ (Table 1).

It is accepted that the sudomotor and cardiovascular adaptations to heat stress are completed within 7–10 days of daily exposure (Garrett et al., 2009; Castle et al., 2011). The typical indicators of heat acclimation, such as reduced exercising HR and T_rectal_ and increased PV and sweat rate were observed in the present study and were similar in magnitude to previous work using an identical heat acclimation protocol (Castle et al., 2011). We are confident therefore that participants attained a heat acclimated state. Mechanistically, an increased vascular filling time mediated by PV expansion is thought to improve cardiovascular stability during exercise-heat stress (Patterson et al., 2004). The observed PV expansion in the present study was maintained until the second HST (+4%, 48 h after the final acclimation session). Although causality cannot be determined, it is feasible that the greater PV in HST2 mediated the increase in exercise SV thereby reducing HR as observed in the HOT group. Additionally, exercise T_rectal_ and PSI were reduced during HST2, likely as a result of the increased sweat rate. While a reduction in exercise heat gain may improve perceptions of exercise difficulty it is unlikely to impact on exercise performance when conditions are compensable (Cheung et al., 2000). Instead, a reduced exercise T_rectal_ induces a leftward shift in the oxyhemoglobin dissociation curve, theoretically enhancing oxygen saturation during exercise (White et al., 2014; Gibson et al., 2015c), which is a more crucial aspect of hypoxic exercise tolerance than maintaining thermal balance in the compensable hypoxic conditions used in the present study. Our results show an improved exercise SpO_2_ in the HOT group post acclimation, occurring alongside greater cardiac stability. Both the reduction in HR, and the reduced blood viscosity (PV expansion) allows for a longer pulmonary system red corpuscle transit time, thereby allowing for a more complete hemoglobin re-saturation (Dempsey et al., 1984). An enhancement in PV prior to an altitude sojourn may also mitigate against the carotid-chemoreceptor dependent diuresis, and subsequent reduced PV and associated reductions in left ventricular filling pressure, stroke volume and cardiac output observed during the initial weeks spent at moderate altitude (Dempsey and Morgan, 2015).

Our results are comparable to those previously observed following a period of isothermic heat acclimation (Gibson et al., 2015c). Isothermic methods of acclimation are suggested to offer a more complete adaptation because session by session workloads are imposed to achieve a target T_rectal_ >38.5°C, thereby maintaining the adaptive stimulus (Fox et al., 1964; Patterson et al., 2004; Taylor and Cotter, 2006; De Castro Magalhães et al., 2010; Gibson et al., 2015a,b,c). However, both fixed work and isothermic models of acclimation have been shown to offer comparable levels of acclimation at a systemic (Gibson et al., 2015b) and gene expression level (Gibson et al., 2015a), indicating each method possesses cross-acclimation and cross-tolerance potential.

We observed no change in absolute $\dot{\text{V}}$O_2_, in addition to a reduction in hypoxic exercise HR 48 h after acclimation, indicating an improvement in gross efficiency as determined from oxygen pulse(O_2_ pulse). Gibson et al. (2015c) noted similar improvements in O_2_ pulse during hypoxic exercise completed 24 h after the final isothermic acclimation session. Together these data illustrate that both fixed load and isothermic acclimation methods induce a heat acclimated phenotype which also induces similar reductions in cardiovascular and thermoregulatory strain upon exposure to subsequent normobaric hypoxic exercise. We observed no post acclimation change in N $\dot{\text{V}}$O_2_ peak in all experimental groups, thus the possibility that improved physiological strain occurred as a result of an improved post heat acclimation $\dot{\text{V}}$O_2_ peak and subsequent reduction in relative exercise intensity can be discounted (Lorenzo et al., 2010). Unfortunately we did not conduct a post-acclimation hypoxic $\dot{\text{V}}$O_2_ peak test, so the role heat acclimation has on hypoxic aerobic capacity could not be determined.

The duration, frequency and total number of intermittent normobaric hypoxic exposures required to acclimate to later normobaric hypoxia is unclear. Our present data indicates that while SpO_2_ was increased during exercise in parallel with a decrease in exercising HR on day 10 of acclimation, full hypoxic acclimation was unlikely to have been achieved. The time course required to achieve a more complete adaptation to normobaric hypoxia may therefore require either additional exposures, or an extended daily hypoxic exercise duration. For example, intermittent hypobaric hypoxic exposures have reported a 2–3% increase in exercise SpO_2_, a 9-20 bpm drop in heart rate, and a 150–160 mL drop in $\dot{\text{V}}$O_2_, and a 6.1 min (16% improvement) in TT performance following 7 daily 4-h resting exposures (Beidleman et al., 2008). In contrast, the same authors reported no change in performance following a matched experimental approach utilizing normobaric methods (Beidleman et al., 2009). The discrepancy in results was attributed to a loss of ventilatory acclimation during the 60 h period between the last acclimation session and follow up testing (Beidleman et al., 2009). In the present study it is possible a loss of ventilatory adaptation occurred during the 48 h period between the last acclimation session and second HST, accounting for the lack of improved physiological tolerance during HST2. The results suggest that heat acclimation offers a more persistent and time efficient means of improving cardiac stability during subsequent normobaric hypoxic exercise. Furthermore, this was attained at a lower level of metabolic strain compared to when the same absolute exercise intensity was conducted in normobaric hypoxia. The optimal duration and frequency required to elicit adaptation to normobaric hypoxia requires further study to enable additional comparisons between environments.

### Heat and hypoxic cross-tolerance

Our data show that a 10 day period of fixed-work exercise acclimation in both heat and hypoxic conditions enhances basal mHSP72 (Figure 2B). Interestingly, the magnitude of mHSP72 accumulation was similar between HYP and HOT despite the greater total physiological strain accrued during heat acclimation (Figure 2C). The time course of HSP72 accumulation throughout a period of heat or hypoxic acclimation has not been studied. Therefore, it cannot be determined whether the different levels of physiological strain observed between conditions in the early phase of acclimation leads to a more rapid or more gradual induction of protective cellular processes. An enhanced reserve of HSP72 is one of the hallmarks of cross tolerance observed in rodent models (Maloyan et al., 1999; Horowitz, 2007; Horowitz and Robinson, 2007). In humans, acclimation to both heat and hypoxia has been shown to elicit increases in basal HSP72 (Mcclung et al., 2008; De Castro Magalhães et al., 2010; Taylor et al., 2011, 2012) suggesting cross-tolerance potential exists. Increased post exercise mHSP72 is likely mediated by an increase in thermal and physiological strain in conditions of heat stress (Lee et al., 2014; Périard et al., 2015), and a transient increase in oxidative stress under hypoxic stress (Taylor et al., 2011, 2012). The results from the HOT group are similar to those reported by others using either fixed workload (Yamada et al., 2007; Mcclung et al., 2008; Hom et al., 2012), or isothermic heat acclimation methods (De Castro Magalhães et al., 2010). Additionally, we observed increases in resting mHSP72 following HYP acclimation, a response also previously observed following 10 daily resting exposures to a similar magnitude of hypoxia (Taylor et al., 2012). However, we are unable to determine whether the increase in mHSP72 observed following hypoxic acclimation was a result of hypoxia *per-se*, and the known increases in oxidative stress that occur in such conditions, or whether the increased relative work-load experienced in HYP was the main driver of the enhanced basal mHSP72. The physiological and cellular strain induced in our control group was not sufficient to induce any changes in mHSP72, which may be due to no substantial exercise induced changes in T_rectal_, nor any exercise induced increase in oxidative stress.

It is well-established that the regulation of HSP synthesis is dependent on the levels existing within the cell (Kregel, 2002). Consequently, prior to acclimation we observed an inverse relationship between basal mHSP72 and the magnitude of post exercise increase in this protein. After acclimation this relationship was no longer present as a result of the increased presence of mHSP72 within the cell. Under non-stressed conditions HSP72 is bound to HSF1. When the cell is exposed to one of the myriad of stressors that require HSP72 chaperone function, HSP72 binds to denatured proteins, freeing HSF1 to migrate to the nucleus and bind with the heat shock element (HSE). More HSP72 protein is then transcribed, and continues to bind with denatured proteins until equilibrium is restored. Excess HSP72 then binds with the HSF1 and transcription is halted (Morimoto, 1998). Therefore, as acclimation progresses and basal HSP72 is accumulated, the cell becomes more robust to the daily challenge to homeostasis imparted via a fixed model of acclimation. As a result, the stress required to sequester HSP72 from HSF1, to begin further transcription, has to cross a new threshold. It is this mechanism of HSP72 action that makes the constant daily strain imparted by isothermic methods of acclimation an attractive model for imparting cellular tolerance (Taylor and Cotter, 2006; Gibson et al., 2015c). However, as our data show, mHSP72 protein is enhanced after 10 days acclimation using a simple fixed workload model, in agreement with the elegant work of Gibson et al. (2015a,c). Furthermore, the elevated basal mHSP72 persisted for at least 48 h after removal from the heat and hypoxic acclimation stimuli, suggesting achievement of a persistent phenotypic shift toward an acclimated state. Subsequently, basal mHSP72 was higher before HST2 compared to HST1 in both the experimental groups and the post exercise increase in mHSP72 was attenuated in both HOT and HYP. The role of increased cellular tolerance on physiological function requires greater scrutiny, as we show that while both modes of acclimation enhance cellular reserves of mHSP72, only the heat acclimated group demonstrated improved physiological function in later hypoxic exercise. Our data support previous observations pertaining to improved cellular tolerance following heat and hypoxic acclimation (Levi et al., 1993; Maloyan et al., 1999; Taylor et al., 2012; Lee et al., 2015; Gibson et al., 2015c).

HIF-1α, the global regulator of cellular and systemic oxygen homeostasis, has also been suggested to play an important role in heat and hypoxic cross tolerance in rodent models (Maloyan et al., 2001, 2005), with increased concentrations and related gene transcripts observed following heat acclimation (Maloyan et al., 1999). However, the role of HIF-1α during acclimation in humans has not been studied. We show that hypoxic acclimation induced a doubling of HIF-1α in the circulation, an increase that was maintained until the second HST. Subsequently, the post HST2 eHIF-1α response was blunted in HYP. We also show an increase in eHIF-1α after the initial heat acclimation session, suggesting that this pathway may be an important mechanism for both heat acclimation and cross-tolerance in humans. However, we acknowledge that the source, function and relationship eHIF-1α has with intracellular HIF-1α (iHIF-1α) is presently unknown. Therefore, while these results are novel, caution is required in their interpretation. Further research is required to determine the relationship between eHIF-1α and iHIF-1α and other HIF-1α associated genes and circulating markers of hypoxic adaptation (e.g., erythropoietin). Doing so will establish the utility of eHIF-1α as a biomarker of acclimation and cross-acclimation.

### Hypoxic time trial performance before and after acclimation

We show, for the first time, that heat acclimation can improve exercise performance under conditions of acute normobaric hypoxia to levels that were comparable to those observed following hypoxic stress. TT performance may have been enhanced following heat acclimation as a result of greater metabolic efficiency and glycogen sparing during the initial 40-min steady-state pre-load trial (Febbraio et al., 2002; Lorenzo et al., 2010). Unfortunately no measurements of muscle glycogen content were possible in the present investigation. The reduced post exercise BLa in the absence of a change in $\dot{\text{V}}$O_2_ peak or lactate thresholds, in combination with improved exercise efficiency (O_2_ pulse) indicates a more efficient aerobic profile. Heat acclimation is known to reduce BLa concentrations for a given intensity (Young et al., 1985; Febbraio et al., 1994). In the present study, mean exercise blood lactate was lower in HST2 following both heat and hypoxic acclimation. It has been suggested that an increase in plasma volume following heat acclimation may have an effect on BLa via an increase in blood flow through the splanchnic circulation, thereby enhancing lactate removal and delaying accumulation (Lorenzo et al., 2010). It is possible that such an effect during HST2 may have led to glycogen sparing via a reduced rate of glycogenesis prior to the TT, thereby preserving glycogen reserves and facilitating a greater maintenance of power output during the TT. Alternatively, the increased TT performance may have occurred as a result of a learning effect following the multiple exercise sessions. However, we took care to ensure that participants were familiarized to the TT protocol in advance. In addition, we validated the pre-loaded TT in our laboratory prior to testing using participants with similar characteristics (Lee et al., 2015). Finally, if performance changes were a result of a learning effect we would expect to see a similar effect in the control group. Instead it appears that the experimental groups had an altered pacing strategy as a result of the acclimation period, with systematic kilometer by kilometer increases in power output, HR, and physiological strain observed in the HYP group, and an altered starting and finishing strategy adopted by the HOT group (Figure 6).

### Study limitations

The results of the present investigation are relevant only to those individuals with a moderate aerobic capacity and should not be applied to those with elite physiology. However, the maintenance of SpO_2_ following either HOT or HYP acclimation is likely of more importance for more well-trained individuals, as they typically experience reduced hemoglobin saturation due to typically larger cardiac outputs and reduced time for gas exchange at higher work-rates (Powers et al., 1989). However, examining physiological responses to hypoxia following acclimation to heat is of interest to athletes that undergo hypoxic training camps. The potential use of heat acclimation to increase ability to tolerate greater work-rates upon arrival to altitude could allow for the maintenance of training volumes and intensities during the initial sessions. However, the role prior acclimation to heat has on longer term hypoxic adaptation has yet to be explored. Additionally, our data only examined normobaric hypoxia. It is possible that responses under ecologically valid hypobaric hypoxic conditions could be different thus future study is required.

### Wider application of our results

Our data indicate that heat based exercise offers a more efficient systemic acclimation response to hypoxia in the time frame examined than normobaric hypoxic training offers, which may have relevance to athletes and military personnel requiring a time-effective means of increasing work capabilities in conditions of moderate hypoxia. An enhancement in cardiac efficiency following repeated heat exposures may be desirable in military populations or individuals sojourning to moderate altitude for short durations without the means or time to fully acclimatize before completing work tasks. Additionally, implementing a hyperthermic stimulus to elicit cross-acclimation responses can be achieved with little specialist equipment compared to the chambers or sojourns required to enable altitude adaptation.

## Conclusion

We show, for the first time, that heat acclimation can improve exercise performance under conditions of acute normobaric hypoxia to levels that were comparable to those observed following hypoxic acclimation. Fixed work heat acclimation is shown to be an effective intervention when improvements in hypoxic exercise tolerance or endurance performance are required. It was demonstrated that heat acclimation was a more effective and longer term (48 h post acclimation) means of improving systemic hypoxic tolerance, as quantified by exercise HR and SpO_2_, than a matched duration and work period of hypoxic acclimation. Both heat and hypoxic acclimation elicit similar changes at the protein level, with each increasing basal HSP72 and eHIF-1α, along with attenuation of post HST HSP72 and eHIF-1α responses to an acute bout of hypoxic exercise. These data confirm previous findings using isothermic models of acclimation and illustrate that increasing physiological strain via exercise-heat stress is an effective, and simple to administer, intervention for invoking both cross-acclimation and cellular cross-tolerance in humans.

## Author contributions

BL, CT, and RJ conceived the study. BL and AM collected the data and performed the biochemical and data analysis. All authors wrote, reviewed, and approved the manuscript.

### Conflict of interest statement

The authors declare that the research was conducted in the absence of any commercial or financial relationships that could be construed as a potential conflict of interest.
